# Crystal structure of diethyl {2,2,2-tri­chloro-1-[2-(1,3-dioxo-2,3-di­hydro-1*H*-isoindol-2-yl)-4-methyl­pentanamido]­eth­yl}phospho­nate

**DOI:** 10.1107/S2056989018008277

**Published:** 2018-06-08

**Authors:** V. S. Brovarets, O. V. Golovchenko, E. B. Rusanov, J. A. Rusanova

**Affiliations:** a Institute of Bioorganic Chemistry and Petrochemistry, National Academy of, Sciences of Ukraine, 1 Murmanska St., Kyiv 02660, Ukraine; b Institute of Organic Chemistry, National Academy of Sciences of Ukraine, 5, Murmanska St., Kyiv 02660, Ukraine; c Department of Chemistry, Taras Shevchenko National University of Kyiv, 64/13, Volodymyrska Street, Kyiv 01601, Ukraine

**Keywords:** crystal structure, phtalimide derivatives, phospho­rylated compounds

## Abstract

In the title phospho­rylated compound, C_20_H_26_Cl_3_N_2_O_6_P, the phthalimide unit is essentially planar (r.m.s. deviation = 0.0129 Å) and the O atoms of this unit deviate from the mean plane by 0.080 (3) and 0.041 (3) Å. In the crystal, pairs of mol­ecules are linked by N—H⋯O and weak C—H⋯O hydrogen bonds involving the same acceptor atom, forming inversion dimers. In addition, π–π stacking inter­actions between the phthalimide groups, with a centroid–centroid distance of 3.7736 (13) Å, and further weak C—H⋯O hydrogen bonds connect the inversion dimers into columns along [01].

## Abstract 2

The crystal structure of the inter­mediate product of the synthesis of phospho­rylated 5-amino-1,3-oxazol-4-yl­phospho­nic acid derivatives is reported.

## Chemical context   

In early 1950, J. W. Cornforth noted that oxazole derivatives rarely occur in nature and were therefore not promising as new biologically active substances. Studies performed mostly during recent decades have shown that the oxazole ring occurs in a multitude of natural products and it has been widely employed as a component of biologically active compounds in medicinal chemistry (Jin *et al.*, 2006 ▸). Various bacteria and marine organisms produce numerous anti­biotics belonging to the oxazole series (Chamberlin *et al.*, 1977 ▸; Bertram *et al.*, 2001 ▸; Jansen *et al.*,1992 ▸; Moody & Bagley, 1998 ▸). Today, numerous oxazole-based synthetic bioregulators with strong anti­microbial, cytostatic, immune stimulating, neuroleptic, analgesic, and other kinds of biological activity are known (Turchi *et al.*, 1986 ▸; Palmer *et al.*, 2003 ▸). In particular, 5-amino-1,3-oxazole and its derivatives are well recognized for their potent and diverse bioregulation activity. Here we present the crystal structure of the title compound, which is an inter­mediate product of synthesis of phospho­rylated 5-amino-1,3-oxazol-4-yl­phospho­nic acid derivatives.
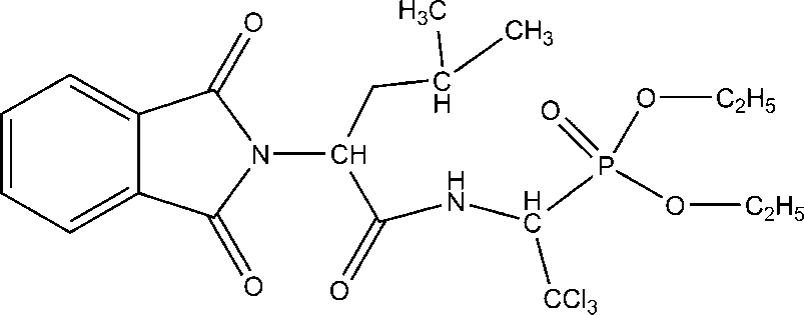


## Structural commentary   

**Figure 1:**
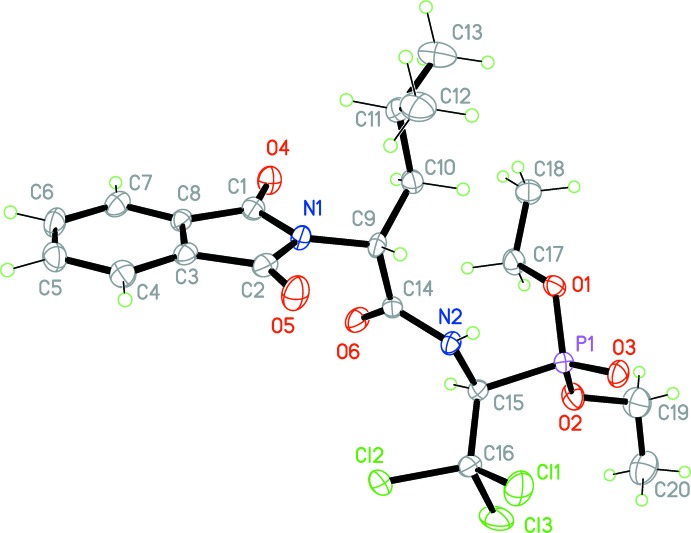
The mol­ecular structure of the title compound with the atom-labelling scheme. Displacement ellipsoids are drawn at the 50% probability level.

The mol­ecular structure of the title compound is illustrated in Fig. 1 ▸. The phthalimide unit (N1/C1–C8) is essentially planar with an r.m.s. deviation of 0.0129 Å. The oxygen atoms O4 and O5 deviate from the mean plane by 0.080 (3) and 0.041 (3) Å, respectively. In the five-membered 3-pyrroline ring, the C—C bond lengths are equivalent [C1—C8 = 1.487 (3) and C2—C3 = 1.486 (3) Å] and the C—N bond lengths differ slightly [N1—C1 = 1.417 (3) and N1—C2 = 1.398 (3) Å], while the corresponding bond angles are not equal [C1—N1—C9 = 127.97 (18) and C2—N1—C9 = 120.53 (17)°] possibly due to the steric influence of the isobutyl group. The mean C—C bond length in the C3–C8 phenyl ring is 1.387 Å. All bond lengths and angles are within normal ranges (Ng, 1992 ▸; Feeder & Jones, 1996 ▸).

In the acetamide moiety, the lone pair of atom N2 is conjugated with the π-system of the C=O group. Thus, the sum of nitro­gen valency angles is 359.3° and the C14—N2 bond length of 1.356 (3) Å is inter­mediate between that for a double and a single bond (1.28 and 1.45 Å, respectively; Allen *et al.*, 1987 ▸). The C15—N2 bond has a typical value for a single bond at 1.442 (3) Å.

The P—O distances in the phospho­nate group show typical values for double [P1=O3 1.4616 (15) Å] and single (with bridging O1 and O2) bonds. The P1—O1 and P1—O2 bonds are equivalent within experimental error with values of 1.5670 (15) and 1.5664 (16) Å, respectively. The C15—P1—O1 and C15—P1—O2 bond angles are equivalent [103.45 (9) and 102.73 (9)°, respectively], while angles O1—P1—O3 and O2—P1—O3 are not [109.82 (9) and 116.77 (9)°], which is probably due to mol­ecular packing effects.

The CCl_3_ group has typical values for the C—Cl distances (the mean C—Cl bond lengths is 1.773 Å). In general, all bonding parameters and the dimensions of the angles in the title complex are in good agreement with those encountered in related complexes (Bhatti *et al.*, 2010 ▸).

## Supra­molecular features   

**Figure 2:**
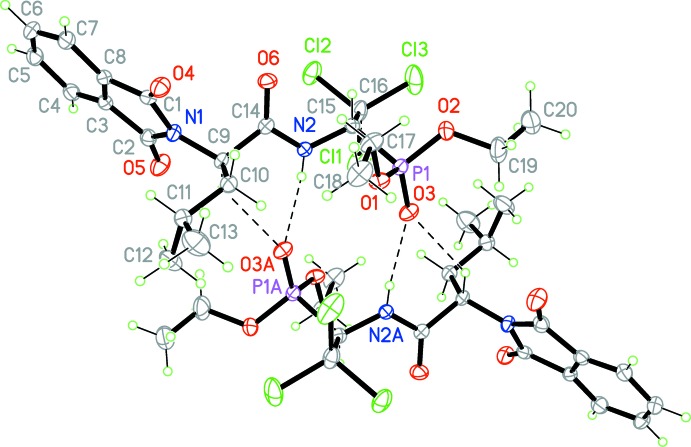
An inversion pair of the title compound, showing the inter­molecular N—H⋯O and C—H⋯O hydrogen bonds [symmetry code: (A) 1 − *x*, 1 − *y*, 1 − *z*].

**Figure 3:**
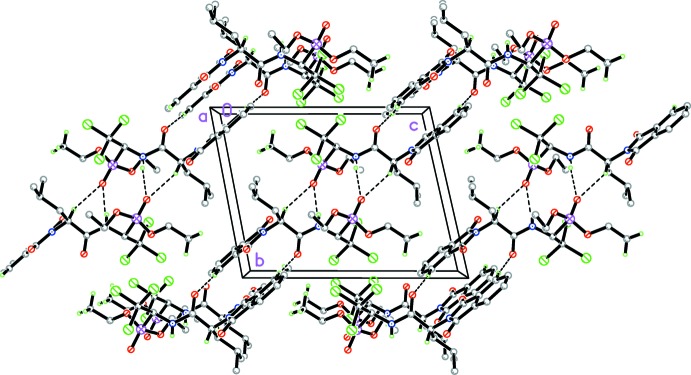
The crystal packing of the title compound viewed along the *a* axis. Inter­molecular N—H⋯O and C—H⋯O hydrogen bonds are shown as dashed lines. Only selected H atoms are shown.

In the crystal, pairs of mol­ecules are linked by N2—H1⋯O3^i^ and C9—H9⋯O3^i^ hydrogen bonds (Table 1 ▸, Fig. 2 ▸) involving the same acceptor atom, forming inversion dimers. In addition, π–π stacking inter­actions between the C3–C8 benzene rings of the phthalimide units connect the dimers into columns along [0
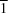
1] with a centroid–centroid distance of 3.7736 (13) Å for *Cg*⋯*Cg*(2 − *x*, −*y*, 2 − *z*). Further weak C—H⋯O hydrogen bonds occur within these columns (Fig. 3 ▸).

## Database survey   

A search of the Cambridge Structural Database (Version 5.38; last update November 2016; Groom *et al.*, 2016 ▸) for related compounds with a phthalimide fragment gave 77 hits including the closely related structures of the 2-[2-(1,3-dioxoisoindolin-2-yl)-acetamido]­acetic acid, (*S*)-4-fluoro-*N*-methyl-2-(1,3-dioxoisoindolin-2-yl)pent-4-enamide di­chloro­methane solvate and (*S*)-4-carbamoyl-4-(1,3-dioxoisoindolin-2-yl)butanoic acid (Bhatti *et al.*, 2010 ▸; Shendage *et al.*, 2004 ▸; Otogawa *et al.*, 2015 ▸). All bond lengths and angles in these related compounds are similar to those in the title compound. Differences in the values of the O—P—O bond angles in the phospho­nate group and C—N—C angles around the pthalimide nitro­gen appear to be due to mol­ecular packing and steric effects.

## Synthesis and crystallization   

The general procedure for the preparation of the title compound was previously described by Lukashuk *et al.* (2015 ▸). A mixture of 2-(1,3-dioxo-2,3-di­hydro-1*H*-isoindol-2-yl)-3-methyl-*N*-(1,2,2,2-tetra­chlo­roeth­yl)butanamide (0.14 mol), triethyl phosphite (30 mL, 0.17 mol), and dry dioxane (150 mL) was refluxed for 3 h. Colourless crystals suitable for single-crystal X-ray analysis were formed after slowly cooling to room temperature. The analytically pure title compound was obtained by solvent evaporation under reduced pressure to dryness (yield 58.84 g, 80% as a yellow oil). Analysis calculated for C_19_H_26_Cl_3_N_2_O_6_P: C, 44.42; H, 4.71; Cl, 20.70; N, 5.45; P, 6.03%; found: C, 44.55; H, 4.86; Cl, 20.82; N, 5.53; P, 6.19%.

The NMR spectra [^1^H (500 MHz), ^31^P (202 MHz), ^13^C (125 MHz); *s*, singlet; *br*, broad; *d*, doublet; *m*, multiplet] were obtained on a Bruker Avance DRX-500 instrument in a solution of DMSO-*d*_6_, relative to inter­nal TMS or external 85% pho­spho­ric acid. ^1^H NMR: 9.32 (½H, *d*, *J* = 9.3 Hz, NH), 9.22 (½H, *d*, *J* = 9.3 Hz, NH), 7.92–7.88 (4H, *m*, aromatic), 5.29–5.21 (1H, *m*, CHP), 4.68–4.61 (1H, *m*, CH), 4.10–4.00 (4H, *m*, 2OCH_2_CH_3_), 2.97–2.90 (1H, *m*, CH), 1.22–1.15 (6H, *m*, 2OCH_2_CH_3_), 1.11–1.05 (3H, *m*, CH_3_), 0.88–0.79 (3H, *m*, CH_3_). ^13^C NMR: 168.61 (*d*, *J* = 4.5 Hz, C=O), 167.41 (*d*, *J* = 4.5 Hz, C=O), 134.41, 134.36, 130.55, 130.53, 122.85, 122.80 (aromatic), 96.21 (*d*, *J* = 14.5 Hz, CCl_3_), 96.06 (*d*, *J* = 14.5 Hz, CCl_3_), 62.30 (*d*, *J* = 6.5 Hz, OCH_2_CH_3_), 62.07 (*d*, *J* = 6.5 Hz, OCH_2_CH_3_), 60.31 (*d*, *J* = 158.8 Hz, CP), 60.26 (*d*, *J* = 158.6 Hz, CP), 59.31, 59.22 (CH), 25.92, 25.84 (CH), 18.55, 18.50 (CH_3_), 18.49, 18.36 (CH_3_), 14.97 (*d*, *J* = 6.0 Hz, OCH_2_CH_3_), 14.86 (*d*, *J* = 6.0 Hz, OCH_2_CH_3_). ^31^P NMR: 14.4. 14.2.

## Refinement   

Crystal data, data collection and structure refinement details are summarized in Table 2 ▸. All C—H hydrogen atoms were placed in calculated positions (C—H = 0.98–1.00Å) and refined in the riding-model approximation with *U*_iso_(H) = 1.2–1.5*U*_eq_(H). The H atom bonded to atom N2 was located in a difference-Fourier map and refined isotropically.

## Supplementary Material

Crystal structure: contains datablock(s) I. DOI: 10.1107/S2056989018008277/lh5875sup1.cif

Structure factors: contains datablock(s) I. DOI: 10.1107/S2056989018008277/lh5875Isup2.hkl

Supporting information file. DOI: 10.1107/S2056989018008277/lh5875Isup3.cml

CCDC reference: 1847170

Additional supporting information:  crystallographic information; 3D view; checkCIF report

## Figures and Tables

**Table 1:** Hydrogen-bond geometry (Å, °)

*D*—H⋯*A*	*D*—H	H⋯*A*	*D*⋯*A*	*D*—H⋯*A*
N2—H1⋯O3^i^	0.76 (2)	2.09 (2)	2.846 (3)	170 (2)
C9—H9⋯O3^i^	1.00	2.47	3.265 (3)	136
C7—H7⋯O6^ii^	0.95	2.46	3.393 (3)	169

Symmetry codes: (i) 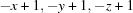; (ii) 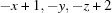.

**Table 2:** Experimental details

Crystal data	
Chemical formula	C_20_H_26_Cl_3_N_2_O_6_P
*M*_r_	527.75
Crystal system, space group	Triclinic, *P* 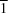
Temperature (K)	173
*a*, *b*, *c* (Å)	8.4601 (2), 10.9425 (3), 13.5321 (4)
α, β, γ (°)	78.188 (2), 88.644 (2), 75.442 (2)
*V* (Å^3^)	1186.32 (6)
*Z*	2
Radiation type	Mo *K*α
μ (mm^−1^)	0.49
Crystal size (mm)	0.24 × 0.19 × 0.08

Data collection	
Diffractometer	Bruker SMART APEXII
Absorption correction	Multi-scan (*SADABS*; Bruker, 2008 ▸)
*T*_min_, *T*_max_	0.87, 0.96
No. of measured, independent and observed [*I* > 2σ(*I*)] reflections	16619, 4423, 3306
*R*_int_	0.052
(sin θ/λ)_max_ (Å^−1^)	0.606

Refinement	
*R*[*F* ^2^ > 2σ(*F* ^2^)], *wR*(*F* ^2^), *S*	0.039, 0.090, 1.08
No. of reflections	4423
No. of parameters	293
H-atom treatment	H atoms treated by a mixture of independent and constrained refinement
Δρ_max_, Δρ_min_ (e Å^−3^)	0.37, −0.44

Computer programs: *APEX2* and *SAINT* (Bruker, 2008 ▸), *SHELXT* (Sheldrick, 2015*a* ▸), *SHELXL2016* (Sheldrick, 2015*b* ▸), *SHELXTL* (Sheldrick, 2008 ▸) and *publCIF* (Westrip, 2010 ▸).

## supplementary crystallographic information

### Crystal data

**Table:**

C_20_H_26_Cl_3_N_2_O_6_P	*Z* = 2
*M**_r_* = 527.75	*F*(000) = 548
Triclinic, *P*1	*D*_x_ = 1.477 Mg m^−^^3^
*a* = 8.4601 (2) Å	Mo *K*α radiation, λ = 0.71073 Å
*b* = 10.9425 (3) Å	Cell parameters from 2780 reflections
*c* = 13.5321 (4) Å	θ = 3.4–45.8°
α = 78.188 (2)°	µ = 0.49 mm^−^^1^
β = 88.644 (2)°	*T* = 173 K
γ = 75.442 (2)°	Plate, colourless
*V* = 1186.32 (6) Å^3^	0.24 × 0.19 × 0.08 mm

### Data collection

**Table:**

Bruker SMART APEXII diffractometer	4423 independent reflections
Radiation source: sealed tube	3306 reflections with *I* > 2σ(*I*)
Graphite monochromator	*R*_int_ = 0.052
φ and ω scans	θ_max_ = 25.5°, θ_min_ = 2.2°
Absorption correction: multi-scan (SADABS; Bruker, 2008)	*h* = −10→9
*T*_min_ = 0.87, *T*_max_ = 0.96	*k* = −13→13
16619 measured reflections	*l* = −16→16

### Refinement

**Table:**

Refinement on *F*^2^	0 restraints
Least-squares matrix: full	Hydrogen site location: mixed
*R*[*F*^2^ > 2σ(*F*^2^)] = 0.039	H atoms treated by a mixture of independent and constrained refinement
*wR*(*F*^2^) = 0.090	*w* = 1/[σ^2^(*F*_o_^2^) + (0.0373*P*)^2^] where *P* = (*F*_o_^2^ + 2*F*_c_^2^)/3
*S* = 1.08	(Δ/σ)_max_ = 0.007
4423 reflections	Δρ_max_ = 0.37 e Å^−^^3^
293 parameters	Δρ_min_ = −0.44 e Å^−^^3^

### Special details

**Table:**

Geometry. All esds (except the esd in the dihedral angle between two l.s. planes) are estimated using the full covariance matrix. The cell esds are taken into account individually in the estimation of esds in distances, angles and torsion angles; correlations between esds in cell parameters are only used when they are defined by crystal symmetry. An approximate (isotropic) treatment of cell esds is used for estimating esds involving l.s. planes.

### Fractional atomic coordinates and isotropic or equivalent isotropic displacement parameters (Å^2^)

**Table:**

	*x*	*y*	*z*	*U*_iso_*/*U*_eq_	
P1	0.38432 (7)	0.33488 (6)	0.45621 (4)	0.01836 (15)	
CL1	0.79937 (8)	0.30683 (7)	0.43532 (5)	0.0447 (2)	
CL2	0.85600 (7)	0.07099 (6)	0.58507 (4)	0.03428 (18)	
CL3	0.69011 (8)	0.09144 (7)	0.39715 (5)	0.0491 (2)	
O1	0.24551 (17)	0.37482 (14)	0.53081 (11)	0.0219 (4)	
O2	0.33202 (19)	0.24320 (15)	0.39667 (11)	0.0267 (4)	
O3	0.42397 (18)	0.45000 (14)	0.39643 (11)	0.0247 (4)	
O4	0.44032 (19)	0.15069 (15)	0.97521 (11)	0.0257 (4)	
O5	0.86366 (19)	0.32359 (16)	0.85338 (12)	0.0310 (4)	
O6	0.54932 (19)	0.11923 (15)	0.74528 (11)	0.0262 (4)	
N1	0.6311 (2)	0.25003 (17)	0.88866 (13)	0.0187 (4)	
N2	0.5722 (2)	0.2870 (2)	0.62148 (13)	0.0182 (4)	
C1	0.5759 (3)	0.1669 (2)	0.96882 (16)	0.0203 (5)	
C2	0.7905 (3)	0.2556 (2)	0.90771 (16)	0.0215 (5)	
C3	0.8451 (3)	0.1656 (2)	1.00570 (15)	0.0194 (5)	
C4	0.9935 (3)	0.1328 (2)	1.05637 (16)	0.0232 (5)	
H4	1.080114	0.170025	1.030700	0.028*	
C5	1.0114 (3)	0.0426 (2)	1.14710 (17)	0.0275 (6)	
H5	1.112112	0.017187	1.184424	0.033*	
C6	0.8833 (3)	−0.0105 (2)	1.18346 (17)	0.0275 (6)	
H6	0.898632	−0.071669	1.245561	0.033*	
C7	0.7331 (3)	0.0230 (2)	1.13198 (16)	0.0248 (6)	
H7	0.645930	−0.013498	1.157508	0.030*	
C8	0.7172 (3)	0.1123 (2)	1.04154 (15)	0.0184 (5)	
C9	0.5453 (3)	0.3201 (2)	0.79368 (15)	0.0188 (5)	
H9	0.599930	0.389770	0.763816	0.023*	
C10	0.3643 (3)	0.3834 (2)	0.80426 (16)	0.0219 (5)	
H10A	0.320062	0.436484	0.737746	0.026*	
H10B	0.305581	0.314522	0.821981	0.026*	
C11	0.3267 (3)	0.4684 (2)	0.88262 (17)	0.0252 (6)	
H11	0.361031	0.412380	0.950673	0.030*	
C12	0.4201 (3)	0.5725 (2)	0.86449 (19)	0.0360 (7)	
H12A	0.537536	0.532229	0.864970	0.054*	
H12B	0.386046	0.630024	0.798851	0.054*	
H12C	0.396886	0.622302	0.917967	0.054*	
C13	0.1436 (3)	0.5270 (3)	0.8824 (2)	0.0418 (7)	
H13A	0.086390	0.458013	0.894246	0.063*	
H13B	0.118619	0.576531	0.936024	0.063*	
H13C	0.107779	0.584253	0.816908	0.063*	
C14	0.5585 (3)	0.2307 (2)	0.71927 (15)	0.0180 (5)	
C15	0.5510 (3)	0.2253 (2)	0.54002 (15)	0.0178 (5)	
H15	0.512342	0.147068	0.570548	0.021*	
C16	0.7154 (3)	0.1776 (2)	0.49071 (16)	0.0269 (6)	
C17	0.1635 (3)	0.2852 (2)	0.59219 (18)	0.0275 (6)	
H17A	0.238670	0.226629	0.646535	0.033*	
H17B	0.127708	0.232492	0.550083	0.033*	
C18	0.0190 (3)	0.3634 (2)	0.63664 (18)	0.0342 (6)	
H18A	−0.039138	0.305605	0.678612	0.051*	
H18B	0.055967	0.414975	0.678234	0.051*	
H18C	−0.054538	0.420814	0.582181	0.051*	
C19	0.2312 (3)	0.2932 (3)	0.30282 (19)	0.0380 (7)	
H19A	0.227578	0.385415	0.277641	0.046*	
H19B	0.118185	0.285406	0.315925	0.046*	
C20	0.3037 (3)	0.2176 (3)	0.22655 (19)	0.0418 (7)	
H20A	0.237677	0.249970	0.163708	0.063*	
H20B	0.415264	0.226244	0.213626	0.063*	
H20C	0.306207	0.126542	0.251793	0.063*	
H1	0.577 (3)	0.357 (2)	0.6099 (17)	0.017 (7)*	

### Atomic displacement parameters (Å^2^)

**Table:**

	*U*^11^	*U*^22^	*U*^33^	*U*^12^	*U*^13^	*U*^23^
P1	0.0179 (3)	0.0187 (3)	0.0166 (3)	−0.0024 (3)	−0.0006 (2)	−0.0022 (2)
CL1	0.0242 (4)	0.0651 (5)	0.0359 (4)	−0.0130 (3)	0.0039 (3)	0.0116 (3)
CL2	0.0240 (3)	0.0416 (4)	0.0269 (3)	0.0092 (3)	−0.0043 (2)	−0.0045 (3)
CL3	0.0398 (4)	0.0655 (5)	0.0372 (4)	0.0148 (4)	−0.0066 (3)	−0.0338 (4)
O1	0.0190 (8)	0.0186 (9)	0.0250 (8)	−0.0024 (7)	0.0047 (7)	−0.0012 (7)
O2	0.0290 (9)	0.0254 (9)	0.0255 (9)	−0.0042 (8)	−0.0092 (7)	−0.0071 (7)
O3	0.0270 (9)	0.0225 (9)	0.0217 (8)	−0.0073 (8)	0.0016 (7)	0.0035 (7)
O4	0.0248 (10)	0.0273 (10)	0.0250 (9)	−0.0107 (8)	−0.0007 (7)	−0.0001 (7)
O5	0.0290 (10)	0.0377 (11)	0.0250 (9)	−0.0150 (9)	0.0010 (7)	0.0046 (8)
O6	0.0407 (11)	0.0176 (9)	0.0199 (8)	−0.0081 (8)	0.0048 (7)	−0.0024 (7)
N1	0.0197 (10)	0.0210 (11)	0.0146 (9)	−0.0060 (9)	−0.0013 (7)	−0.0007 (8)
N2	0.0240 (11)	0.0159 (12)	0.0146 (10)	−0.0062 (10)	−0.0001 (8)	−0.0015 (8)
C1	0.0270 (14)	0.0144 (12)	0.0200 (12)	−0.0052 (11)	0.0004 (10)	−0.0044 (10)
C2	0.0240 (13)	0.0225 (13)	0.0191 (12)	−0.0061 (11)	0.0027 (10)	−0.0061 (10)
C3	0.0249 (13)	0.0168 (12)	0.0172 (11)	−0.0051 (11)	0.0020 (9)	−0.0057 (9)
C4	0.0241 (13)	0.0225 (13)	0.0246 (13)	−0.0064 (11)	−0.0021 (10)	−0.0072 (10)
C5	0.0293 (14)	0.0254 (14)	0.0262 (13)	−0.0015 (12)	−0.0088 (11)	−0.0070 (11)
C6	0.0384 (15)	0.0223 (14)	0.0179 (12)	−0.0038 (12)	−0.0045 (11)	0.0003 (10)
C7	0.0318 (14)	0.0239 (14)	0.0199 (12)	−0.0100 (11)	0.0031 (10)	−0.0041 (10)
C8	0.0237 (13)	0.0161 (12)	0.0154 (11)	−0.0045 (10)	0.0007 (9)	−0.0036 (9)
C9	0.0248 (13)	0.0175 (12)	0.0128 (11)	−0.0055 (10)	−0.0022 (9)	0.0008 (9)
C10	0.0226 (13)	0.0207 (13)	0.0210 (12)	−0.0023 (10)	−0.0027 (9)	−0.0042 (10)
C11	0.0324 (14)	0.0224 (14)	0.0197 (12)	−0.0042 (12)	0.0003 (10)	−0.0054 (10)
C12	0.0431 (17)	0.0289 (15)	0.0406 (16)	−0.0090 (13)	0.0021 (13)	−0.0177 (12)
C13	0.0337 (16)	0.0431 (17)	0.0513 (18)	−0.0047 (14)	0.0105 (13)	−0.0228 (14)
C14	0.0166 (12)	0.0189 (13)	0.0170 (11)	−0.0032 (10)	0.0007 (9)	−0.0016 (10)
C15	0.0194 (12)	0.0176 (12)	0.0158 (11)	−0.0036 (10)	0.0005 (9)	−0.0037 (9)
C16	0.0218 (13)	0.0369 (15)	0.0180 (12)	−0.0002 (11)	0.0011 (10)	−0.0056 (11)
C17	0.0210 (13)	0.0277 (14)	0.0289 (13)	−0.0060 (11)	0.0038 (10)	0.0046 (11)
C18	0.0225 (14)	0.0422 (17)	0.0309 (14)	−0.0018 (12)	0.0076 (11)	−0.0004 (12)
C19	0.0327 (15)	0.0481 (18)	0.0337 (15)	−0.0071 (13)	−0.0117 (12)	−0.0123 (13)
C20	0.0566 (19)	0.0424 (18)	0.0311 (15)	−0.0186 (15)	−0.0055 (13)	−0.0096 (13)

### Geometric parameters (Å, º)

**Table:**

P1—O3	1.4616 (15)	C9—C14	1.526 (3)
P1—O2	1.5564 (16)	C9—C10	1.531 (3)
P1—O1	1.5670 (15)	C9—H9	1.0000
P1—C15	1.839 (2)	C10—C11	1.526 (3)
CL1—C16	1.764 (3)	C10—H10A	0.9900
CL2—C16	1.780 (2)	C10—H10B	0.9900
CL3—C16	1.772 (2)	C11—C12	1.520 (3)
O1—C17	1.454 (3)	C11—C13	1.520 (3)
O2—C19	1.474 (3)	C11—H11	1.0000
O4—C1	1.202 (3)	C12—H12A	0.9800
O5—C2	1.209 (3)	C12—H12B	0.9800
O6—C14	1.220 (2)	C12—H12C	0.9800
N1—C2	1.398 (3)	C13—H13A	0.9800
N1—C1	1.417 (3)	C13—H13B	0.9800
N1—C9	1.459 (2)	C13—H13C	0.9800
N2—C14	1.356 (3)	C15—C16	1.545 (3)
N2—C15	1.442 (3)	C15—H15	1.0000
N2—H1	0.76 (2)	C17—C18	1.496 (3)
C1—C8	1.487 (3)	C17—H17A	0.9900
C2—C3	1.486 (3)	C17—H17B	0.9900
C3—C4	1.374 (3)	C18—H18A	0.9800
C3—C8	1.387 (3)	C18—H18B	0.9800
C4—C5	1.394 (3)	C18—H18C	0.9800
C4—H4	0.9500	C19—C20	1.480 (4)
C5—C6	1.389 (3)	C19—H19A	0.9900
C5—H5	0.9500	C19—H19B	0.9900
C6—C7	1.392 (3)	C20—H20A	0.9800
C6—H6	0.9500	C20—H20B	0.9800
C7—C8	1.387 (3)	C20—H20C	0.9800
C7—H7	0.9500		
			
O3—P1—O2	116.77 (9)	C13—C11—H11	108.1
O3—P1—O1	109.82 (9)	C10—C11—H11	108.1
O2—P1—O1	107.96 (9)	C11—C12—H12A	109.5
O3—P1—C15	115.02 (10)	C11—C12—H12B	109.5
O2—P1—C15	102.73 (9)	H12A—C12—H12B	109.5
O1—P1—C15	103.45 (9)	C11—C12—H12C	109.5
C17—O1—P1	123.88 (14)	H12A—C12—H12C	109.5
C19—O2—P1	121.74 (15)	H12B—C12—H12C	109.5
C2—N1—C1	111.45 (18)	C11—C13—H13A	109.5
C2—N1—C9	120.53 (17)	C11—C13—H13B	109.5
C1—N1—C9	127.97 (18)	H13A—C13—H13B	109.5
C14—N2—C15	121.5 (2)	C11—C13—H13C	109.5
C14—N2—H1	118.0 (17)	H13A—C13—H13C	109.5
C15—N2—H1	119.8 (17)	H13B—C13—H13C	109.5
O4—C1—N1	125.4 (2)	O6—C14—N2	122.8 (2)
O4—C1—C8	129.3 (2)	O6—C14—C9	122.46 (19)
N1—C1—C8	105.22 (18)	N2—C14—C9	114.63 (19)
O5—C2—N1	124.7 (2)	N2—C15—C16	111.14 (18)
O5—C2—C3	128.9 (2)	N2—C15—P1	107.70 (14)
N1—C2—C3	106.43 (19)	C16—C15—P1	116.87 (14)
C4—C3—C8	122.5 (2)	N2—C15—H15	106.9
C4—C3—C2	129.6 (2)	C16—C15—H15	106.9
C8—C3—C2	107.91 (19)	P1—C15—H15	106.9
C3—C4—C5	117.0 (2)	C15—C16—CL1	111.57 (16)
C3—C4—H4	121.5	C15—C16—CL3	110.61 (16)
C5—C4—H4	121.5	CL1—C16—CL3	109.04 (12)
C6—C5—C4	120.6 (2)	C15—C16—CL2	108.95 (14)
C6—C5—H5	119.7	CL1—C16—CL2	108.53 (13)
C4—C5—H5	119.7	CL3—C16—CL2	108.04 (13)
C5—C6—C7	122.2 (2)	O1—C17—C18	107.44 (19)
C5—C6—H6	118.9	O1—C17—H17A	110.2
C7—C6—H6	118.9	C18—C17—H17A	110.2
C8—C7—C6	116.6 (2)	O1—C17—H17B	110.2
C8—C7—H7	121.7	C18—C17—H17B	110.2
C6—C7—H7	121.7	H17A—C17—H17B	108.5
C7—C8—C3	121.0 (2)	C17—C18—H18A	109.5
C7—C8—C1	130.0 (2)	C17—C18—H18B	109.5
C3—C8—C1	108.93 (18)	H18A—C18—H18B	109.5
N1—C9—C14	110.41 (17)	C17—C18—H18C	109.5
N1—C9—C10	114.38 (17)	H18A—C18—H18C	109.5
C14—C9—C10	108.24 (17)	H18B—C18—H18C	109.5
N1—C9—H9	107.9	O2—C19—C20	108.7 (2)
C14—C9—H9	107.9	O2—C19—H19A	109.9
C10—C9—H9	107.9	C20—C19—H19A	109.9
C11—C10—C9	115.53 (18)	O2—C19—H19B	109.9
C11—C10—H10A	108.4	C20—C19—H19B	109.9
C9—C10—H10A	108.4	H19A—C19—H19B	108.3
C11—C10—H10B	108.4	C19—C20—H20A	109.5
C9—C10—H10B	108.4	C19—C20—H20B	109.5
H10A—C10—H10B	107.5	H20A—C20—H20B	109.5
C12—C11—C13	111.1 (2)	C19—C20—H20C	109.5
C12—C11—C10	111.88 (19)	H20A—C20—H20C	109.5
C13—C11—C10	109.45 (19)	H20B—C20—H20C	109.5
C12—C11—H11	108.1		
			
O3—P1—O1—C17	172.13 (16)	N1—C1—C8—C3	1.9 (2)
O2—P1—O1—C17	43.80 (18)	C2—N1—C9—C14	97.2 (2)
C15—P1—O1—C17	−64.61 (18)	C1—N1—C9—C14	−79.8 (3)
O3—P1—O2—C19	−37.1 (2)	C2—N1—C9—C10	−140.4 (2)
O1—P1—O2—C19	87.18 (18)	C1—N1—C9—C10	42.6 (3)
C15—P1—O2—C19	−163.92 (17)	N1—C9—C10—C11	51.7 (3)
C2—N1—C1—O4	174.9 (2)	C14—C9—C10—C11	175.29 (19)
C9—N1—C1—O4	−7.8 (4)	C9—C10—C11—C12	54.9 (3)
C2—N1—C1—C8	−2.6 (2)	C9—C10—C11—C13	178.5 (2)
C9—N1—C1—C8	174.64 (19)	C15—N2—C14—O6	9.9 (3)
C1—N1—C2—O5	−176.7 (2)	C15—N2—C14—C9	−166.63 (18)
C9—N1—C2—O5	5.8 (3)	N1—C9—C14—O6	39.6 (3)
C1—N1—C2—C3	2.2 (2)	C10—C9—C14—O6	−86.3 (2)
C9—N1—C2—C3	−175.22 (17)	N1—C9—C14—N2	−143.88 (19)
O5—C2—C3—C4	−3.4 (4)	C10—C9—C14—N2	90.2 (2)
N1—C2—C3—C4	177.7 (2)	C14—N2—C15—C16	−108.9 (2)
O5—C2—C3—C8	178.0 (2)	C14—N2—C15—P1	121.90 (19)
N1—C2—C3—C8	−0.9 (2)	O3—P1—C15—N2	71.35 (17)
C8—C3—C4—C5	−0.2 (3)	O2—P1—C15—N2	−160.69 (14)
C2—C3—C4—C5	−178.7 (2)	O1—P1—C15—N2	−48.41 (16)
C3—C4—C5—C6	−0.1 (3)	O3—P1—C15—C16	−54.52 (19)
C4—C5—C6—C7	0.1 (4)	O2—P1—C15—C16	73.43 (18)
C5—C6—C7—C8	0.3 (3)	O1—P1—C15—C16	−174.29 (16)
C6—C7—C8—C3	−0.6 (3)	N2—C15—C16—CL1	−62.8 (2)
C6—C7—C8—C1	179.4 (2)	P1—C15—C16—CL1	61.4 (2)
C4—C3—C8—C7	0.6 (3)	N2—C15—C16—CL3	175.64 (15)
C2—C3—C8—C7	179.4 (2)	P1—C15—C16—CL3	−60.2 (2)
C4—C3—C8—C1	−179.39 (19)	N2—C15—C16—CL2	57.0 (2)
C2—C3—C8—C1	−0.6 (2)	P1—C15—C16—CL2	−178.83 (11)
O4—C1—C8—C7	4.5 (4)	P1—O1—C17—C18	−169.00 (15)
N1—C1—C8—C7	−178.1 (2)	P1—O2—C19—C20	133.89 (19)
O4—C1—C8—C3	−175.5 (2)		

### Hydrogen-bond geometry (Å, º)

**Table:**

*D*—H···*A*	*D*—H	H···*A*	*D*···*A*	*D*—H···*A*
N2—H1···O3^i^	0.76 (2)	2.09 (2)	2.846 (3)	170 (2)
C9—H9···O3^i^	1.00	2.47	3.265 (3)	136
C7—H7···O6^ii^	0.95	2.46	3.393 (3)	169

Symmetry codes: (i) −*x*+1, −*y*+1, −*z*+1; (ii) −*x*+1, −*y*, −*z*+2.
